# Is Decellularized Porcine Small Intestine Sub-mucosa Patch Suitable for Aortic Arch Repair?

**DOI:** 10.3389/fped.2018.00149

**Published:** 2018-05-30

**Authors:** Antonio F. Corno, Paul Smith, Laurynas Bezuska, Branko Mimic

**Affiliations:** ^1^East Midlands Congenital Heart Centre, University Hospitals of Leicester, Leicester, United Kingdom; ^2^Cardiovascular Research Center, University of Leicester, Leicester, United Kingdom

**Keywords:** aortic coarctation, aortic arch anomalies, aortic arch surgery, patch materials, aorta

## Abstract

**Introduction:** We reviewed our experience with decellularized porcine small intestine sub-mucosa (DPSIS) patch, recently introduced for congenital heart defects.

**Materials and Methods:** Between 10/2011 and 04/2016 a DPSIS patch was used in 51 patients, median age 1.1 months (5 days to 14.5 years), for aortic arch reconstruction (45/51 = 88.2%) or aortic coarctation repair (6/51 = 11.8%). All medical records were retrospectively reviewed, with primary endpoints interventional procedure (balloon dilatation) or surgery (DPSIS patch replacement) due to patch-related complications.

**Results:** In a median follow-up time of 1.5 ± 1.1 years (0.6–2.3years) in 13/51 patients (25.5%) a re-intervention, percutaneous interventional procedure (5/51 = 9.8%) or re-operation (8/51 = 15.7%) was required because of obstruction in the correspondence of the DPSIS patch used to enlarge the aortic arch/isthmus, with median max velocity flow at Doppler interrogation of 4.0 ± 0.51 m/s. Two patients required surgery after failed interventional cardiology. The mean interval between DPSIS patch implantation and re-intervention (percutaneous procedure or re-operation) was 6 months (1–17 months). While there were 3 hospital deaths (3/51 = 5.9%) not related to the patch implantation, no early or late mortality occurred for the subsequent procedure required for DPSIS patch interventional cardiology or surgery. The median max velocity flow at Doppler interrogation through the aortic arch/isthmus for the patients who did not require interventional procedure or surgery was 1.7 ± 0.57 m/s.

**Conclusions:** High incidence of re-interventions with DPSIS patch for aortic arch and/or coarctation forced us to use alternative materials (homografts and decellularized gluteraldehyde preserved bovine pericardial matrix).

## Introduction

Aortic coarctation is quite frequently associated with aortic arch hypoplasia, requiring attention at the time of surgery. The criteria generally agreed to define the presence of aortic arch hypoplasia are: (a) aortic arch size mm < body weight kg + 1; (b) aortic arch diameter z-score < −2.0; (c) ratio of transverse arch diameter to descending aorta <50%.

Surgery for aortic coarctation with aortic arch reconstruction can be performed with various surgical approaches. Despite good outcomes reported with the technique of resection of the aortic coarctation and end-to-end anastomosis extended to the aortic arch through a postero-lateral thoracotomy (1–5), the currently preferred option is repair through median sternotomy with cardiopulmonary bypass. The approach with cardiopulmonary bypass through median sternotomy, necessary when associated heart malformations require surgical treatment in the same session, can be accomplished with various techniques of aortic arch reconstruction with or without the use of a patch enlargement.

After experimental (6–8) and clinical (9–11) studies, the decellularized porcine small intestine sub-mucosa (DPSIS) patch has been introduced with the commercial name of CorMatrix® (CorMatrix Cardiovascular, Roswell, GA) for the surgical treatment of congenital heart defects, with implantations performed for closure of septal defects, valve repair, and as vascular patch in both the systemic and pulmonary circulations (12–16).

Despite the initial positive comments, in the last few years several reports appeared with less than favorable medium and long-term outcomes (17–27).

We therefore decided to review our experience with DPSIS patch implantation.

## Materials and methods

Between October 2011 and April 2016, in 51 patients, median age 1.1 months (range 5 days to 14.5 years) and median weight 4.0 kg (range 2.2–50.2 kg), a DPSIS was used for either the reconstruction of the aortic arch (45/51 = 88.2%) or the repair of aortic coarctation (6/51 = 11.8%). All the initial operations have been performed by two surgeons, using exactly the same surgical technique for aortic arch enlargement.

In this group of 51 patients there were 19 patients (19/51 = 37%) with associated malformations: ten patients with ventricular septal defect (VSD), three neonates with hypoplastic left heart syndrome (HLHS), two patients with severe left ventricular outflow tract obstruction (LVOTO), two neonates with aortic arch interruption (AAI), one of whom with associated Transposition of the Great Arteries (TGA), one with complete atrio-ventricular septal defect (cAVSD) and one with congenitally corrected TGA.

All medical records were retrospectively reviewed, with primary endpoints being either interventional procedure (balloon dilatation) or surgery (DPSIS patch replacement or augmentation) due to the recurrent aortic arch/isthmus obstruction.

In all patients the consent for surgery and for the utilization of data for publication and/or presentation in scientific meetings was signed by one of the parents or by the legal guardian the day before surgery.

The retrospective study was approved by the Ethical Committee of the Children's Hospital, University Hospital of Leicester.

## Results

While there were 3 hospital deaths (3/51 = 5.9%) not related to the patch implantation, no early or late mortality occurred for the subsequent procedure required for DPSIS patch interventional cardiology or surgery.

Among the 19 patients with associated malformations, in the same surgical procedure with aortic arch/isthmus reconstruction, 6 neonates underwent pulmonary artery banding (four with VSD, one with AVSD and one with congenitally corrected TGA), three had a Norwood procedure (HLHS), two a Damus-Kaye-Stansel procedure (severe LVOTO), and one arterial switch (TGA).

**Figure 1:**
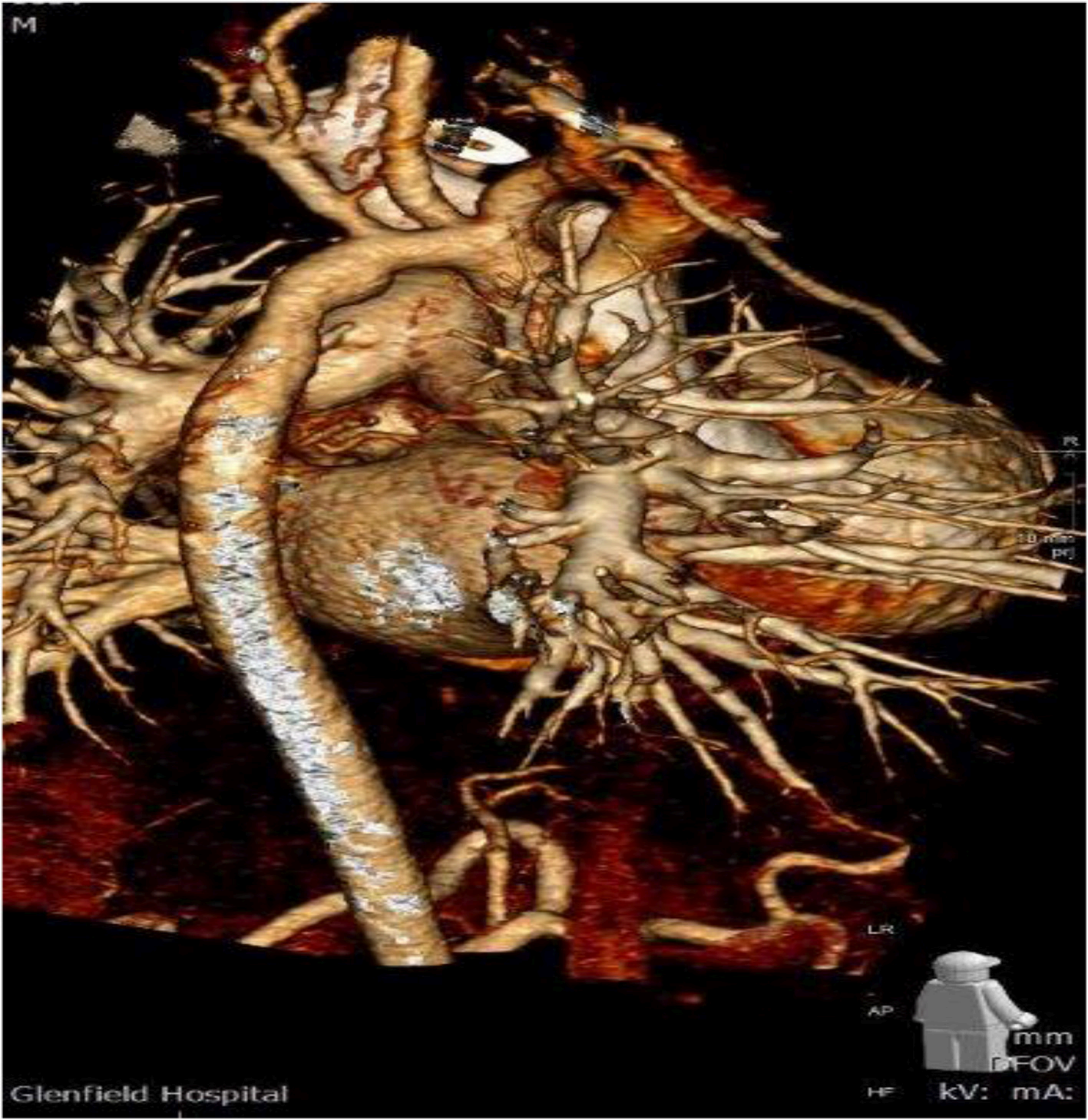
CT scan with 3D reconstruction showing the hypoplastic aortic arch two and half year after DPSIS patch arch reconstruction.

In a median follow-up time of 1.5 ± 1.1 years (range 7 months to 2.3 years) in 13/51 patients (25.5%), a re-intervention, either percutaneous interventional cardiology procedure (5/51 = 9.8%) or re-operation (8/51 = 15.7%) was required because of obstruction in the correspondence of the DPSIS patch used for enlargement of the aortic arch/isthmus. In these 13 patients the indication for re-intervention was given because of a max velocity flow at Doppler interrogation through the aortic arch/isthmus was recorded with a median value of 4.0 ± 0.51 m/s. In all these patients, after the demonstration of the presence of significant pressure gradient at Doppler, the morphology of the recurrent narrowing in the correspondence of the aortic arch/isthmus, shown with echocardiography, was confirmed with either cardiac CT scan or MRI with 3D reconstruction (Figure 1).

The 11 patients who required re-intervention (percutaneous procedure or re-operation) and the 2 waiting for surgical re-intervention had the DPSIS patch implantation at a median age of 8 days (range 4 days to 8 years). The initial diagnosis and procedure and the type of re-intervention are listed in Table 1.

**Table 1:** AA, aortic arch; HLHS, hypoplastic left heart syndrome; PAB, pulmonary artery banding; TGA, transposition of the great arteries; VSD, ventricular septal defect.

**Patient**	**Initial diagnosis**	**Associated lesions**	**Initial operation**	**Re-intervention 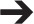 **	
1	Hypoplastic AA	VSD	AA patch enlargement, VSD closure	Ballon dilatation
2	Hypoplastic AA	VSD	AA patch enlargement, VSD closure	Surgery
3	Hypoplastic AA	Multiple VSDs	AA patch enlargement, PAB	Ballon dilatation
4	Hypoplastic AA	Multiple VSDs	AA patch enlargement, PAB	Surgery
5	Hypoplastic AA	HLHS	AA patch enlargement, Norwood procedure	Ballon dilatation
6	Hypoplastic AA	Univentricular heart	AA patch enlargement, bidirectional Glenn	Ballon dilatation
7	Hypoplastic AA	Shone complex	AA patch enlargement, mitral valve repair	Surgery
8	Hypoplastic AA	Supravalvular aortic stenosis	AA patch enlargement, ascending aorta reconstruction	Ballon dilatation
9	Hypoplastic AA		AA patch enlargement	Surgery
10	Hypoplastic AA		AA patch enlargement	Surgery
11	Hypoplastic AA		AA patch enlargement	Waiting for surgery
12	Hypoplastic AA	VSD	AA patch enlargement, VSD closure	Waiting for surgery
13	AA interruption	TGA, VSD	AA patch reconstruction, arterial switch, VSD closure	Surgery

The mean interval between the original surgery and the re-intervention was 6 months (range 10 days to 17 months). Two among these patients required surgery after failed attempt with interventional cardiology procedure.

No early and late mortality occurred for the DPSIS patch surgical implantation and also for the subsequent interventional procedure or surgery.

The max velocity flow at Doppler interrogation through the aortic arch/isthmus for the patients who did not require interventional procedure or surgery was recorder with a mean value of 1.7 ± 0.57 m/s.

No difference has been found between the group of patients who underwent re-intervention because aortic arch/isthmus obstruction and the patients who didn't require re-intervention, as the two groups were homogeneous for age, body weight, associated congenital heart defects and morphology of the aortic arch/isthmus.

No difference has been found between the two surgeons in relationship to the incidence of re-intervention.

In particular, the correlation between the geometrical morphology of the aortic arch and the incidence of re-interventions was investigated in relationship to the presence of gothic aortic arch (28–31). Despite the presence of gothic aortic arch was observed in the pre-operative CT scan investigation in 2/13 (= 15.4%) patients requiring re-intervention vs. only 2/38 (= 5.3%) patients who didn't require re-intervention, the difference didn't reach statistical difference at the *T*-test (*P* = 0.12).

**Figure 2:**
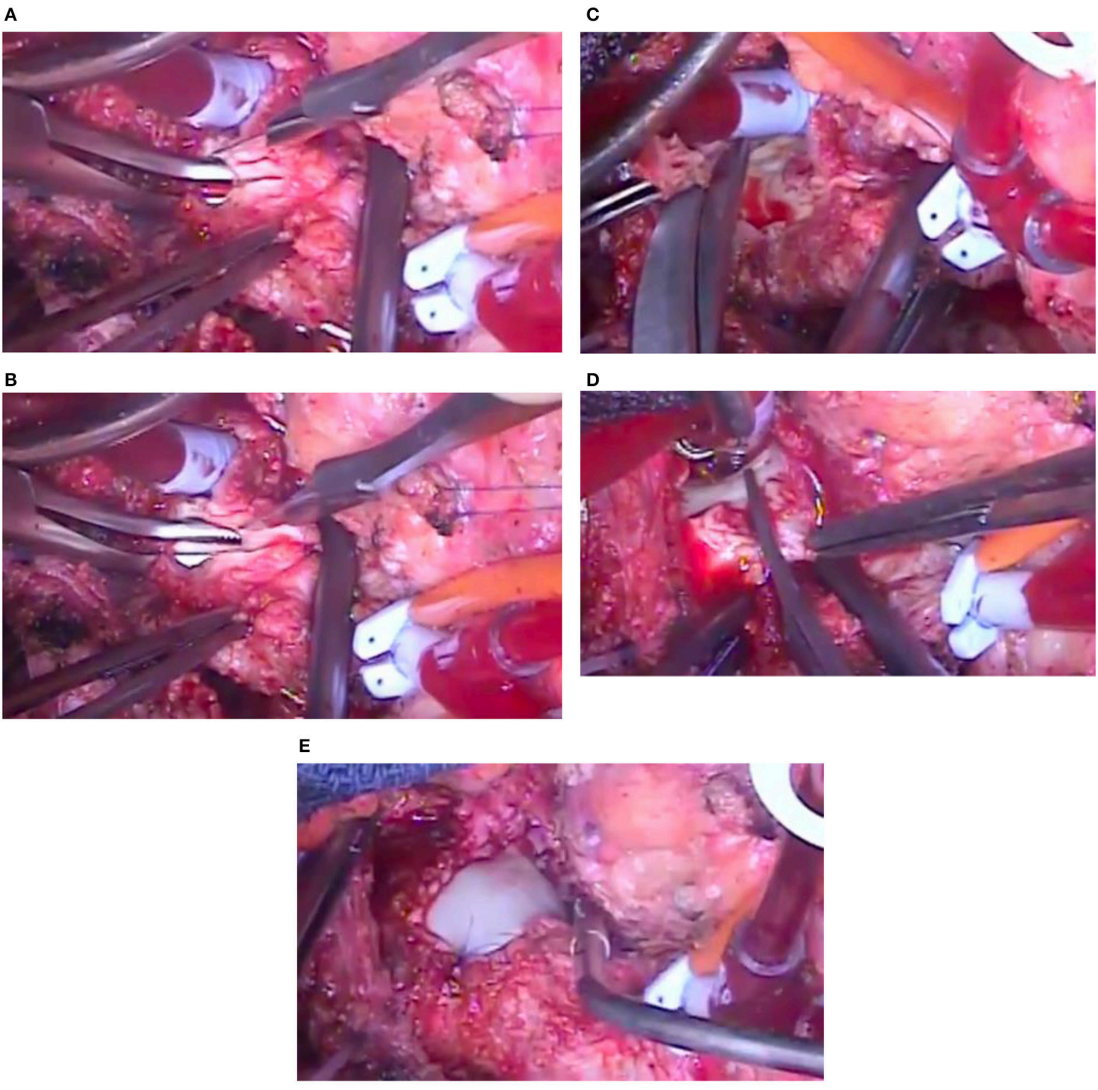
Intra-operative images of the knife incision of the patch, extended with the introduction of a surgical instrument (coming from the left side) through the opening in the narrowing of the aortic arch **(A,B)**, removal by scissors of the thick patch superiorly **(C)** and inferiorly **(D)** and new aortic arch enlargement with another patch material **(E)** in the same patient of Figure 1.

The surgically explanted DPSIS patches, macroscopically very thick and rigid (Figure 2), underwent histologic evaluations, with these reports: (a) fragments of fibrous tissue containing cellular necrotic tissue surrounded by histiocytes and occasional multinucleate giant cells; (b) fibrous thickening with neovascularization; (c) presence of perivascular lymphocytes and plasma cells.

## Discussion

Surgery for aortic arch reconstruction with aortic coarctation can be performed with various surgical approaches for cardiopulmonary bypass. Deep hypothermia and circulatory arrest (32–34) has been progressively replaced by regional cerebral perfusion (35–38) with or without associated myocardial perfusion and beating heart (39, 40), and more recently with distal aortic cannulation for lower body perfusion (41–43). Our standard approach was to use regional cerebral and myocardial perfusion with aortic cross clamping and myocardial ischemia only for the repair of the associated intra-cardiac defects, but we do not consider this matter relevant for the surgical results related to this study.

The aortic arch reconstruction can be accomplished with different surgical techniques avoiding the use of a patch to enlarge the aortic narrowing: the ascending sliding arch aortoplasty and the aortic arch advancement technique are the most used (36, 44–47). Alternatively a patch is utilized to enlarge the aortic arch narrowing, with various synthetic and biological materials reported as patch enlargement, including polytetrafluoroethylene, aortic or pulmonary homografts, autologous or heterologous pericardium, autologous vascular patch from the pulmonary artery or the aorta (29, 37, 48–51). The advent of bioengineering has certainly expanded the horizon of materials potentially available as biological patch (52).

Recurrent obstruction in the correspondence of the aortic arch and/or isthmus, requiring either interventional procedures (balloon dilatation/stent) or surgery, is the most frequently reported complication, with an extremely variable incidence reported from 2 to 38%. The recurrent obstruction seems to be correlated with the surgical technique utilized, with the lowest incidence of re-intervention reported with ascending sliding arch aortoplasty and/or aortic arch advancement technique (2–3%), in comparison with the patch enlargement technique (18–38%), occasionally correlated with the patch material used (22, 29–31, 37, 45–51, 53).

In our unit the patch enlargement technique was introduced years ago, motivated by the desire to avoid the potential risk of left bronchus compression, and remained the first choice in the period of the current study, despite very low incidence (0.7 and 1.8%) of left bronchial compression respectively reported with the aortic advancement technique (46) and the end-to side anastomosis (47). With regard to the left bronchial compression surgical approaches have also been suggested either for the prevention of the complication, such as the anterior translocation of the right pulmonary artery (54), or for the treatment, such as the autograft aortic arch extension and sleeve resection (55).

The DPSIS patch was introduced in our unit on October 2011 for closure of septal defects, valve repair, and as vascular patch in both the systemic and pulmonary circulations after positive experimental (6) and clinical reports (12). The company (CorMatrix Cardiovascular, Roswell, GA) claimed, in the official advertising of the patch, the ability of the DPSIS patch to regrow and remodel, constituting a structurally support gradually replaced by native tissues, leaving no foreign material behind, encouraging the body's natural immune response, allowing capillary in-growth and infiltration of white blood cells into newly remodeled tissue, and avoiding inflammation and scarring (CorMatrix®).

Unfortunately the expectations have not been followed by the clinical evidence, with several clinical reports showing poor medium-term clinical outcomes (17–27).

In addition several histologic examinations of the explanted DPSIS patches showed an intense, predominantly eosinophilic inflammatory response with rapid degenerative changes and developing fibrosis (18, 20–22) and a high rate of intimal hyperplasia formation (19), not different from our observations; also aneurysm formation of the patch has been reported after aortic arch implantation (22).

Even in more contrast with the advantages claimed by the company, in no cases the histologic examination of the explanted DPSIS patches showed evidence of tissue integration or recellularization, with the patch acting as a bioscaffold for reconstitution of the native heart tissue ingrowth (20, 23, 27).

Our experience with the histology of the explanted patches confirmed the observations reported in the literature.

### Limits of the study

We are aware of the limits of our study, in particular:

- this is a single center retrospective analysis. As a matter of fact, when the DPSIS patch has been introduced in our unit a prospective data collection was not started.
- the results obtained with DPSIS patch have not been compared with other patch materials because the alternative patches have been used either in the years before or after this experience, and therefore the duration of follow-up would have been completely different, and would have not allowed a meaningful comparison.
- the results obtained with DPSIS patch have not been compared with other surgical techniques because the techniques reported in the discussion, the ascending sliding arch aortoplasty and the aortic arch advancement technique, have been introduced only recently, and therefore the number of patients and follow-up are limited.

## Conclusion

The high incidence of re-interventions with DPSIS patch for aortic arch and/or coarctation observed in our experience (25.5%) forced us to abandon the use of this material and replace it with alternative biological materials: homografts and decellularized gluteraldehyde preserved bovine pericardial matrix (CardioCel®, Admedus, Perth, Australia) (56). The preliminary results seem favorable, but a larger number of patients and longer follow-up will be required for a meaningful comparison with the DPSIS patch.

## Author contributions

AC provided a substantial contribution to the conception and design of the work, drafted the work and revised for the content, and agreed to be accountable for all aspects of the work. PS gave an important contribution to the acquisition and analysis of the data of the work. LB helped for the acquisition and analysis of the data of the work, as well as to the interpretation of the data. BM contributed to the interpretation of the data of the work, as well as the revision and approval of the content before submission for publication.

### Conflict of interest statement

The authors declare that the research was conducted in the absence of any commercial or financial relationships that could be construed as a potential conflict of interest. The reviewer MC and handling Editor declared their shared affiliation.
